# Bioinformatics-Driven Identification of Ferroptosis-Related Gene Signatures Distinguishing Active and Latent Tuberculosis

**DOI:** 10.3390/genes16060716

**Published:** 2025-06-18

**Authors:** Rakesh Arya, Hemlata Shakya, Viplov Kumar Biswas, Gyanendra Kumar, Sumendra Yogarayan, Harish Kumar Shakya, Jong-Joo Kim

**Affiliations:** 1 Department of Biotechnology, Yeungnam University, Gyeongsan 38541, Republic of Korea; rakesharya101@yu.ac.kr; 2 Department of Biomedical Engineering, Shri G. S. Institute of Technology and Science, Indore 452003, Madhya Pradesh, India; hemlata.shakya19@gmail.com; 3 Department of Cell Biology and Molecular Genetics, University of Maryland, Campus Drive, College Park, MD 20742, USA; viplov@umd.edu; 4 Department of IoT and Intelligent Systems, Manipal University Jaipur, Jaipur 303007, Rajasthan, India; gyanendra.kumar@jaipur.manipal.edu; 5 Faculty of Information Science and Technology (FIST), Multimedia University (MMU), Ayer Keroh 75450, Malaysia; 6 Department of Artificial Intelligence and Machine Learning, Manipal University Jaipur, Jaipur 303007, Rajasthan, India; harish.shakya@jaipur.manipal.edu

**Keywords:** ferroptosis, tuberculosis, immune response, biomarkers, gene expression

## Abstract

Background: Tuberculosis (TB) remains a major global public health challenge, and diagnosing it can be difficult due to issues such as distinguishing active TB from latent TB infection (LTBI), as well as the sample collection process, which is often time-consuming and lacks sensitivity and specificity. Ferroptosis is emerging as an important factor in TB pathogenesis; however, its underlying molecular mechanisms are not fully understood. Thus, there is a critical need to establish ferroptosis-related diagnostic biomarkers for tuberculosis (TB). Methods: This study aimed to identify and validate potential ferroptosis-related genes in TB infection while enhancing clinical diagnostic accuracy through bioinformatics-driven gene identification. The microarray expression profile dataset GSE28623 from the Gene Expression Omnibus (GEO) database was used to identify ferroptosis-related differentially expressed genes (FR-DEGs) associated with TB. Subsequently, these genes were used for immune cell infiltration, Gene Set Enrichment Analysis (GSEA), functional enrichment and correlation analyses. Hub genes were identified using Weighted Gene Co-expression Network Analysis (WGCNA) and validated in independent datasets GSE37250, GSE39940, GSE19437, and GSE31348. Results: A total of 21 FR-DEGs were identified. Among them, four hub genes (*ACSL1*, *PARP9*, *TLR4*, and *ATG3*) were identified as diagnostic biomarkers. These biomarkers were enriched in immune-response related pathways and were validated. Immune cell infiltration, GSEA, functional enrichment and correlation analyses revealed that multiple immune cell types could be activated by FR-DEGs. Throughout anti-TB therapy, the expression of the four hub gene signatures significantly decreased in patients cured of TB. Conclusions: In conclusion, ferroptosis plays a key role in TB pathogenesis. These four hub gene signatures are linked with TB treatment effectiveness and show promise as biomarkers for differentiating TB from LTBI.

## 1. Introduction

Tuberculosis (TB), caused by *Mycobacterium tuberculosis* (*Mtb*), remains a leading cause of death globally, surpassing COVID-19 in 2023. The World Health Organization’s “Global Tuberculosis Report 2024”, indicates 10.8 million new TB cases globally in 2023, with an incidence of 134 per 100,000 population [1]. Among these new cases, 662,000 (6.1%) were co-infected with HIV and 400,000 (3.7%) were patients with multidrug-resistant (MDR) or rifampicin-resistant (RR) TB. Among new cases, the rate of multidrug-resistant/rifampicin-resistant TB (MDR/RR-TB) was 3.2% and 16% among previously treated cases. TB claimed 1.25 million lives in 2023, which makes TB the leading cause of death from a single infectious disease globally, with the number of deaths nearly twice the number of HIV/AIDS-associated deaths [1]. Latent TB infection (LTBI) affects approximately one-third of the world’s population, with a 5–10% lifetime risk of developing active TB. There are various tests, including an interferon-γ release assay (IGRA) and the tuberculin skin test (TST), which are used to diagnose LTBI but not active TB [2]. Diagnosing TB remains challenging, highlighting the need for new blood-based biomarkers.

*Mtb* enters the lungs and infects alveolar macrophages by invading the host’s immune response and manipulating cellular machinery [3]. Cell death plays a critical role in the progression of latent TB to active TB. Most studies indicate that apoptosis, pyroptosis, and autophagy inhibit pathogen survival, while necrosis and ferroptosis promote *Mtb* replication and dissemination [4]. However, recent research suggests that apoptosis can also facilitate *Mtb* survival and release [5], suggesting that the immune outcomes of host cell death modes in a given situation may be multifaceted. Ferroptosis, a non-programmed cell death characterized by intracellular iron accumulation and lipid peroxidation, leads to cell membrane rupture [6,7]. The probable role of ferroptosis in the *Mtb*-mediated induction of cell death involves the multiplication of *Mtb* within host macrophages, which can contribute to intracellular iron accumulation, increased mitochondrial superoxide, and lipid peroxidation inducing necrotic cell death. Particularly, *Mtb*-infected macrophages exhibit elevated glutathione peroxidase 4 (GPX4) expression and glutathione (GSH) levels, both associated with ferroptotic death [8]. Therefore, ferroptosis plays a significant role in TB pathogenesis, and ferroptosis-related genes (FRGs) may influence TB development.

Multiple studies have identified gene signatures that both uncover the mechanisms of disease and act as innovative biomarkers for differentiating active TB from LTBI [8,9,10]. Recent research combines high-throughput RNA sequencing with bioinformatics tools to pinpoint differentially expressed genes (DEGs) and critical pathways driving TB pathogenesis [11,12,13,14,15]. Previous studies have implicated several issues, including the identification of effective biomarkers for prognosis, diagnosis, and treatment efficacy; the understanding of key metabolic pathways that can be targeted; and the elucidation of molecular mechanisms such as ferroptosis, underlying host cell death in relation to *Mtb* infections to develop effective interventions.

**Figure 1:**
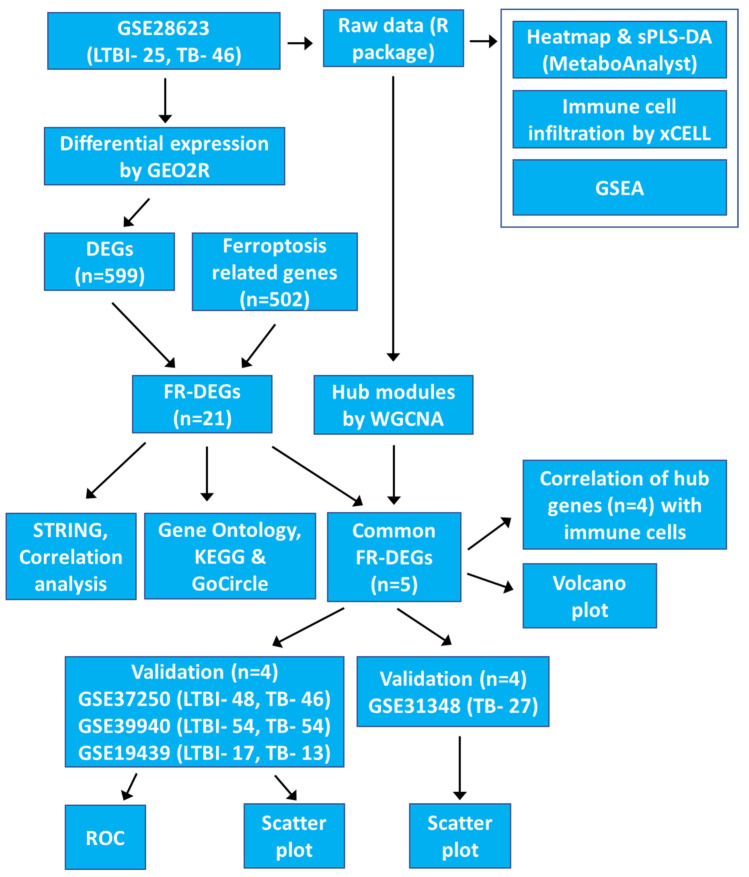
The flowchart outlines the gene expression analysis pipeline for the GSE28623 dataset, highlighting differential expression, ferroptosis-related genes, hub modules, and validation steps.

This study analyses gene expression profiles from TB patients using publicly available data from the Gene Expression Omnibus (GEO) database. The aim is to identify ferroptosis-related hub genes that regulate ferroptosis associated with TB infection. We identified four ferroptosis-related hub genes, *ACSL1*, *PARP9*, *TLR4*, and *ATG3*, as potential biomarkers for TB. We also demonstrated that these genes can activate multiple immune cell types through various pathways related to ferroptosis and TB, using immune cell infiltration, GSEA, functional enrichment and correlation analyses. Our study successfully established a link between gene expression levels and the effectiveness of anti-TB treatment, concluding that the four hub gene signatures hold promise for distinguishing TB from LTBI. The protocol and algorithms used are outlined in Figure 1.

## 2. Materials and Methods

### 2.1. Data Collection and Processing

The gene expression datasets of GSE28623, GSE37250, GSE39940, GSE19439, and GSE31348 were downloaded from the Gene Expression Omnibus (GEO) database (https://www.ncbi.nlm.nih.gov/geo/ accessed on 18 April 2024). These datasets were selected because they investigated the blood transcriptional expression profiles in patients with TB and LTBI. GSE28623, designated as the discovery set, included 71 whole blood samples from 45 TB patients and 25 individuals with LTBI, based on the GPL4133 platform (Agilent-014850 Whole Human Genome Microarray 4x44 K G4112F) [16]. GSE37250 [17] and GSE39940 [18], designated as validation sets, were based on the GPL10558 platform (Illumina HumanHT-12 V4.0 expression beadchip). GSE37250 contained whole blood samples from 46 TB patients and 48 individuals with LTBI. GSE39940 contained whole blood samples from 54 TB patients and 54 individuals with LTBI. GSE19439 [19], another validation set, included 13 samples from TB patients and 17 samples from individuals with LTBI, based on the GPL6947 platform (Illumina HumanHT-12 V3.0 expression beadchip).

GSE31348 [20], the fourth validation set, contained ex vivo blood samples analyzed from 27 first-episode pulmonary TB patients both before initiating standard therapy and at multiple time points following successful treatment. At the time of patient admission, 27 samples were collected prior to anti-TB treatment administration. After the commencement of treatment, an additional 27 samples were collected each week at weeks 1, 2, 4, and 26, resulting in a total of 135 samples throughout the study. The dataset was based on the GPL570 platform (Affymetrix Human Genome U133 Plus 2.0 Arrays).

### 2.2. Data Processing and Statistical Analysis

Raw data matrix files, including microarray annotations, were downloaded from the National Center for Biotechnology Information (NCBI). The data was analyzed using MetaboAnalyst (version 5.0; https://www.metaboanalyst.ca, accessed on 18 April 2024), a web-based platform designed for comprehensive omics data analysis. Specifically, heatmaps and sparse Partial Least Squares Discriminant Analysis (sPLS-DA) plots were generated to visualize and analyze the data. To ensure data consistency and comparability, quantile normalization and auto-scaling were applied to normalize and scale the data.

### 2.3. Immune Cell Infiltration Analysis Using xCell

xCell, a web-based tool, was employed to perform cell type enrichment analysis of gene expression data for 64 immune and stromal cell types (https://comphealth.ucsf.edu/app/xcell, accessed on 6 May 2024). This method utilizes a gene signature-based algorithm, trained on thousands of pure cell types from diverse sources, to accurately represent tissue expression profiles [21]. The xCell algorithm was utilized to quantify changes in the proportions of infiltrating immune cells within the immune microenvironment, comparing TB and LTBI samples. The results were visualized using boxplots (*p*-value < 0.05) and barplots.

### 2.4. Gene Set Enrichment Analysis (GSEA)

Gene Set Enrichment Analysis (GSEA) is a computational approach used to determine if predefined sets of genes exhibit statistically significant, concordant differences between two biological states, such as different phenotypes. In this study, GSEA was performed using version 4.3.2 of the GSEA software. The analysis utilized the WikiPathways subset within the Molecular Signature Database (MsigDB), which encompasses 791 annotated gene sets [22,23].

### 2.5. Differential Gene Expression Analysis Using GEO2R

Gene expression analysis was conducted using GEO2R, an NCBI-built web tool (https://www.ncbi.nlm.nih.gov/geo/geo2r, accessed on 18 April 2024) [24], to identify differentially expressed genes (DEGs) between TB and LTBI groups. The *p*-value was adjusted using the Benjamini–Hochberg false discovery rate (FDR) method and automated data transformation with limma precision weights (vooma) and normalization was applied. Rows without gene symbols and duplicate genes were excluded. Statistically significant DEGs from the GSE28623 datasets were selected based on filtering criteria of log_2_ (fold change) ≥ ±1 and *p*-value < 0.01.

### 2.6. Ferroptosis-Related Genes, Protein Interaction, and Correlation Analysis

Ferroptosis-related genes (FRGs) were downloaded from the FerrDb database, which includes experimentally validated ferroptosis drivers, suppressors, markers, and ferroptosis–disease associations [25]. Specifically, 502 FRGs were downloaded from the FerrDb database (http://www.zhounan.org/ferrdb, accessed on 18 April 2024). A Venn diagram was generated using the identified DEGs (n = 599) and the FRGs (n = 502) to determine the combined FR-DEGs. The STRING database (https://www.string-db.org, accessed on 13 May 2024) was used to construct a protein–protein interaction (PPI) network of the identified FR-DEGs, with a minimum required interaction score confidence of 0.5 [26]. Correlation analysis of 21 FR-DEGs was performed using SRplot (https://www.bioinformatics.com.cn, accessed on 26 April 2024) with a significance threshold of *p*-value < 0.05 [27].

### 2.7. Pathway and Functional Annotation of FR-DEGs

Gene Ontology (GO) enrichment analysis of FR-DEGs–including biological processes (BP), cellular components (CC), and molecular functions (MF) and Kyoto Encyclopedia of Genes and Genomes (KEGG) pathway analysis were performed using ShinyGO v0.82 [28]. FR-DEGs were analyzed based on their presence in specific pathways, −log_10_ (FDR), and fold enrichment. GO terms with a *p*-value < 0.05 were deemed significantly enriched. To further analyze FR-DEGs, a GOCircle plot was generated using the GOplot package (1.0.2) in R. this plot utilized WikiPathways data from the STRING database, based on logFC and z-score, to identify the top 10 pathways involving the DE-FRGs.

### 2.8. Weighted Gene Co-Expression Network Analysis (WGCNA)

WGCNA was employed to identify highly relevant gene modules associated with TB [29]. The WGCNA was performed using TBtools-II (version 2.210) [30]. Gene expression profile data was processed using expected counts with variance stabilizing transformation (VST) normalization. The data underwent two filtering steps: first, the removal of low gene counts based on a sample percentage of 0.9 and an expression cutoff of 10 and second, filtering using median absolute deviation (MAD). A soft threshold power (β) of 7 was set at R^2^ = 0.8. Average linkage hierarchical clustering was performed to group genes with similar expression patterns into modules. Gene dendrograms were generated with a minimum module size of 30 and a cuttree height of 0.3. Hub genes were selected based on module correlation and gene significance of active TB.

### 2.9. Venn Diagram and Volcano Plot Analysis of Hub Genes

To identify hub genes, a Venn diagram was created with yellow module genes (n = 226) and FR-DEGs (n = 21). The five hub genes were highlighted in a volcano plot, with cutoffs of log_2_ (fold change) ≥ ±1 and a *p*-value < 0.05. The volcano plot was generated using the VolcaNoseR online tool (https://huygens.science.uva.nl/VolcaNoseR, accessed on 20 November 2024) [31]. The *GALNT14* gene was not used for further analysis.

### 2.10. Correlation Analysis Between Hub Genes with Immune Cell Infiltration

The correlation analysis between the expression of hub genes (n = 4) and immune cell infiltration within the GSE28623 dataset was conducted using the “ggplot2” package in R. The results of this analysis were then visualized in the form of a lollipop plot [32].

### 2.11. Validation and Diagnostic Potential of Hub Genes

The expression levels of four hub genes were validated across four independent GEO datasets: GSE37250, GSE39940, GSE19439, and GSE31348. Scatter plots, displaying the median with interquartile range, PCA plots, and heatmaps were generated for each dataset. The *GALNT14* gene was excluded from validation due to its absence in the first three validation sets. Each FR-DEG was evaluated as a potential diagnostic biomarker of TB using Receiver Operating Characteristic (ROC) plots generated by SRplot.

## 3. Results

### 3.1. Gene Expression Analysis and Group Clustering

**Figure 2:**
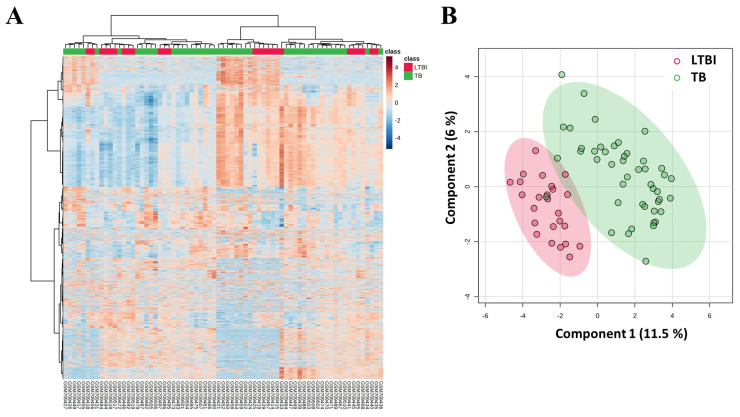
Heatmaps and sPLS-DA analysis of GSE28623 dataset. (**A**) The heatmap displayed gene expression profiles with hierarchical clustering, distinguishing LTBI (red) and TB (green) samples based on expression levels. (**B**) The sPLS-DA plot showed the overlapping of LTBI (red) and TB (green) groups, illustrating variability between the two conditions.

The gene expression analysis of the GSE28623 dataset, which included 45,015 features, was visualized using a heatmap. The heatmap visualizes features or genes through color intensity, where an increase toward dark red or more positive values signifies upregulation, while a decrease toward dark blue or more negative values indicates downregulation. This heatmap highlighted potential overlapping molecular profiles rather than distinct separation due to several reasons such as sample heterogeneity, the biological overlap of molecular signatures, immune status, etc. The sPLS-DA plot visually represents how samples are grouped and separated based on their features (variables) in relation to a target variable (groups or classes). Additionally, a sPLS-DA plot was generated, showing overlapping clusters corresponding to LTBI and TB groups (Figure 2). The low variance observed in sPLS-DA may be attributed to shared immune responses and disease pathophysiology between LTBI and TB groups, leading to cluster overlap.

### 3.2. Changes in Immune Characteristics Between LTBI and TB Groups

**Figure 3:**
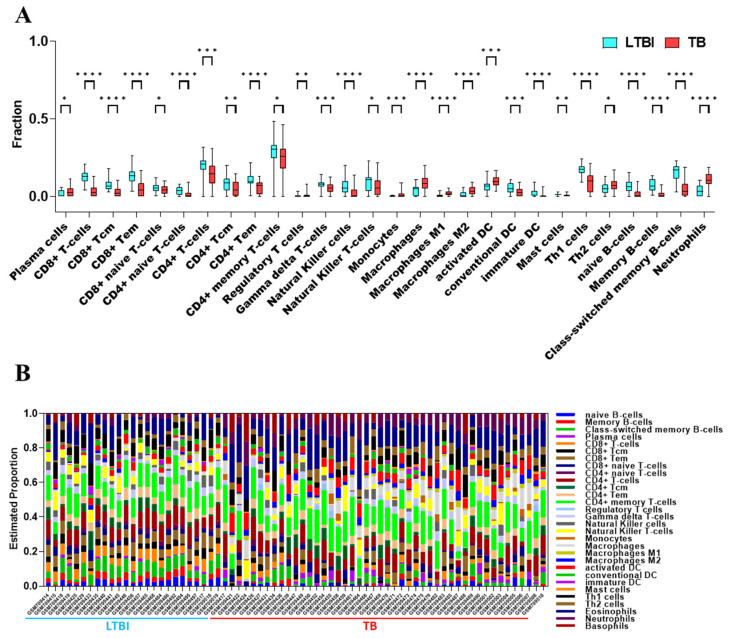
Changes in immune cell characteristics between LTBI and TB groups in GSE28623 dataset. (**A**) Boxplot compares the fractions of immune cell types in LTBI and TB groups, with significant differences marked by asterisks. * *p* < 0.05, ** *p* < 0.01, *** *p* < 0.001, **** *p* < 0.0001. (**B**) Barplot illustrates the estimated proportions of immune cells across individual LTBI and TB samples.

The xCell analysis revealed distinct immune profile differences between LTBI and TB patients, with TB patients exhibiting higher proportions of innate immune cells such as monocytes, macrophages (M1 and M2), neutrophils, and dendritic cells, while showing lower proportions of adaptive immune cells, including CD4+ and CD8+ T-cell subsets, Natural Killer cells, and B-cell populations (Figure 3A). Additionally, the study identified correlations between different immune cell ratios, demonstrating how infection impacts immune composition, providing insights into the immune response dynamics of tuberculosis (Figure 3B).

### 3.3. Gene Set Enrichment Analysis (GSEA) and Ferroptosis Correlation with TB

**Figure 4:**
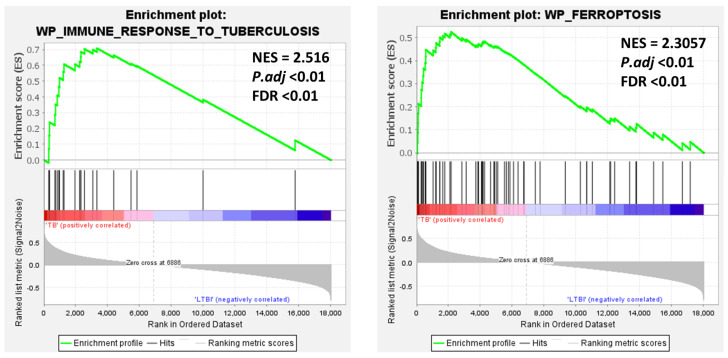
Gene Set Enrichment Analysis (GSEA) in the GSE28623 dataset. The enrichment plots illustrate the “immune response to tuberculosis” and “ferroptosis” pathways analyzed in the GSE28623 dataset, with high normalized enrichment scores (NES) of 2.516 and 2.3057, respectively, and significant *P.adj* and FDR values, indicating their strong association with TB pathogenesis.

The GSEA results revealed that the plots of immune response to tuberculosis and ferroptosis were positively correlated with the TB group. Specifically, most genes in the blood samples of TB patients were enriched in tuberculosis (NES = 2.516, p-adj < 0.01) and ferroptosis (NES = 2.305, p-adj < 0.01). This indicates a higher inflammatory response in the context of ferroptosis in pulmonary TB (Figure 4). These results suggest that ferroptosis plays a significant role in the development of tuberculosis.

### 3.4. Identification of DEGs Using GEO2R

Through GEO2R analysis, 599 differentially expressed genes (DEGs) were identified in the GSE28623 dataset, with 371 genes upregulated and 228 genes downregulated.

### 3.5. Identification, Interaction, and Correlation Analysis of FR-DEGs in TB

**Figure 5:**
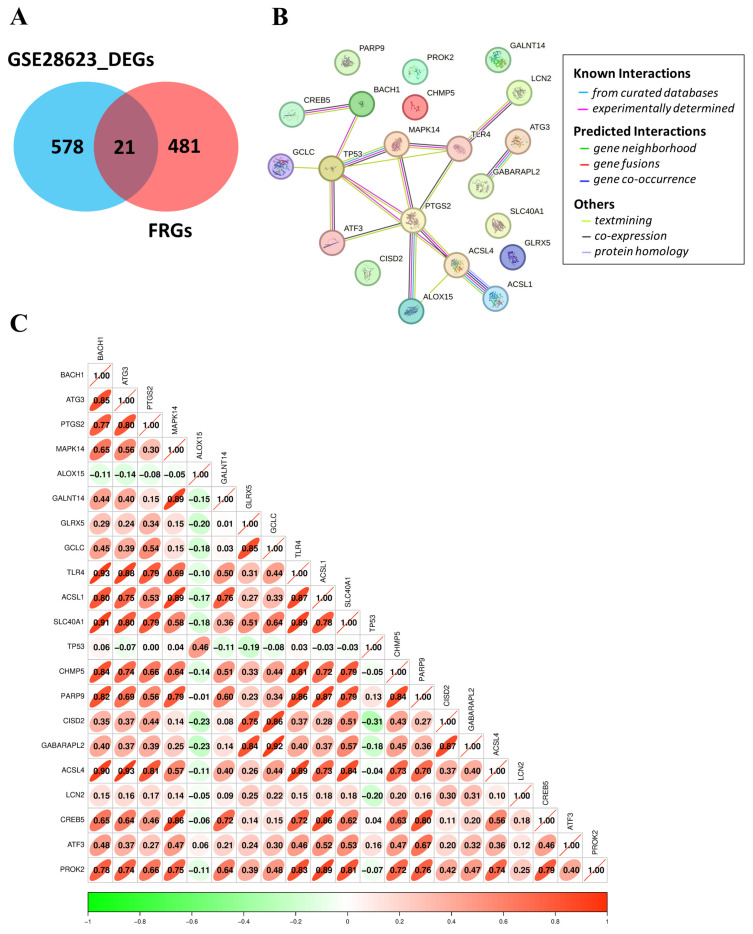
Identification of analysis of FR-DEGs: (**A**) Venn diagram depicting overlapped genes between DEGs and FRGs. (**B**) STRING showing the interaction of 21 FR-DEGs. (**C**) The correlation analysis represents the degree of correlation between the 21 FR-DEGs.

Exploring ferroptosis mechanisms in tuberculosis infection, 502 FRGs were obtained from the FerrDb database. The intersection of 599 DEGs and 502 FRGs resulted in the identification of 21 FR-DEGs: *BACH1*, *ATG3*, *PTGS2*, *MAPK14*, *ALOX15*, *GALNT14*, *GLRX5*, *GCLC*, *TLR4*, *ACSL1*, *SLC40A1*, *TP53*, *CHMP5*, *PARP9*, *CISD2*, *GABARAPL2*, *ACSL4*, *LCN2*, *CREB5* *ATF3*, and *PROK2* (Figure 5A and Table 1). These 21 FR-DEGs were further analyzed for their interaction using the STRING database, revealing significant interactions among them (Figure 5B). Correlation analysis was conducted to examine the relationships among these FR-DEGs (Figure 5C). Significant positive correlations were observed: *ACSL1* with *TLR4* (r = 0.93, *p*-value < 0.05), *ATG3* (r = 0.75, *p*-value < 0.05), and *PARP9* (r = 0.87, *p*-value < 0.05). *TLR4* also showed significant positive correlation with *ATG3* (r = 0.88, *p*-value < 0.05) and *PARP9* (r = 0.86, *p*-value < 0.05). Also, *ATG3* presented a significantly positive correlation with *PARP9* (r = 0.69, *p*-value < 0.05).

### 3.6. Pathway and Functional Annotations of FR-DEGs

**Figure 6:**
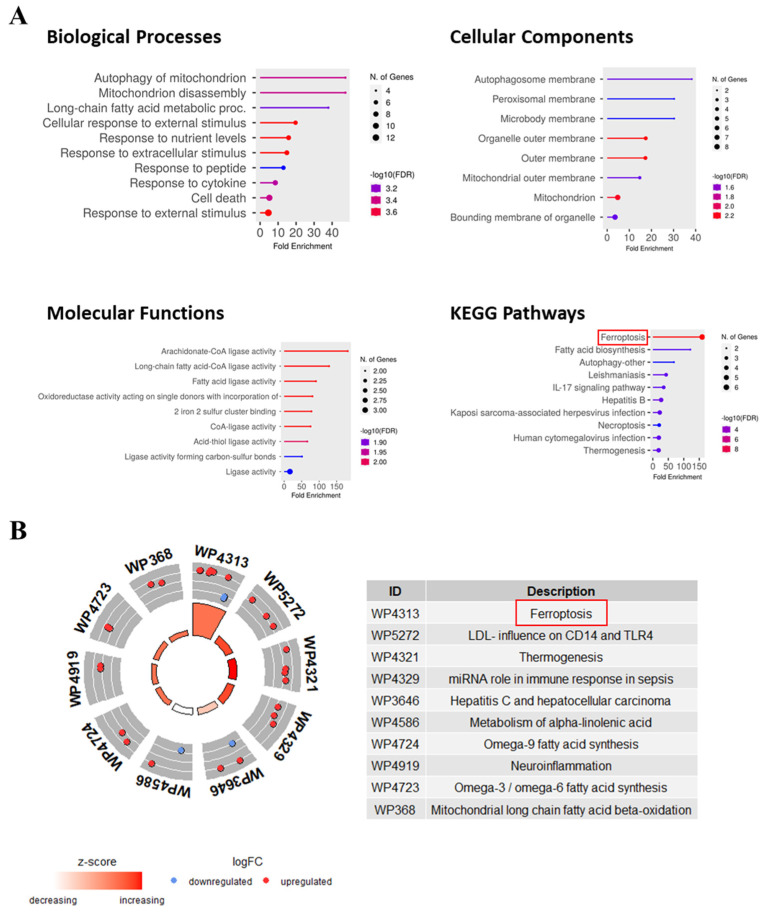
Functional enrichment, pathway, and GOCircle analysis of 21 FR-DEGs. (**A**) The lollipop chart describes the GO and KEGG enrichment analysis, highlighting significant biological processes, cellular components, molecular functions, and pathways associated with TB and ferroptosis. (**B**) WikiPathway analysis of FR-DEGs based on logFC values and z-score showed the top 10 most important pathways including ferroptosis, emphasizing their potential involvement in TB pathogenesis and immune regulation.

To explore the functions of FR-DEGs, GO, KEGG, and WikiPathway analyses were performed. GO biological process (BP) analysis revealed enrichment in the autophagy of mitochondria, long-chain fatty acid metabolic processes, the cellular response to external stimulus, the response to cytokines, etc. Cellular component (CC) analysis highlighted enrichment in the autophagosome, peroxisomal enzyme, microbody membrane, etc. Molecular functions (MF) showed enrichment in arachidonate–CoA ligase activity, long-chain fatty acids–CoA ligase activity, fatty acid ligase activity, etc. KEGG pathway analysis revealed enrichment in ferroptosis, fatty acid biosynthesis, autophagy, etc. (Figure 6A). GOCircle analysis using WikiPathways identified ferroptosis as a highly important pathway with a high z-score and a significant number of FR-DEGs. These findings suggest that FR-DEGs likely play an essential role in the regulating the immune response related to tuberculosis (Figure 6B).

### 3.7. Identification of Key Modules Related to TB Using WGCNA

**Figure 7:**
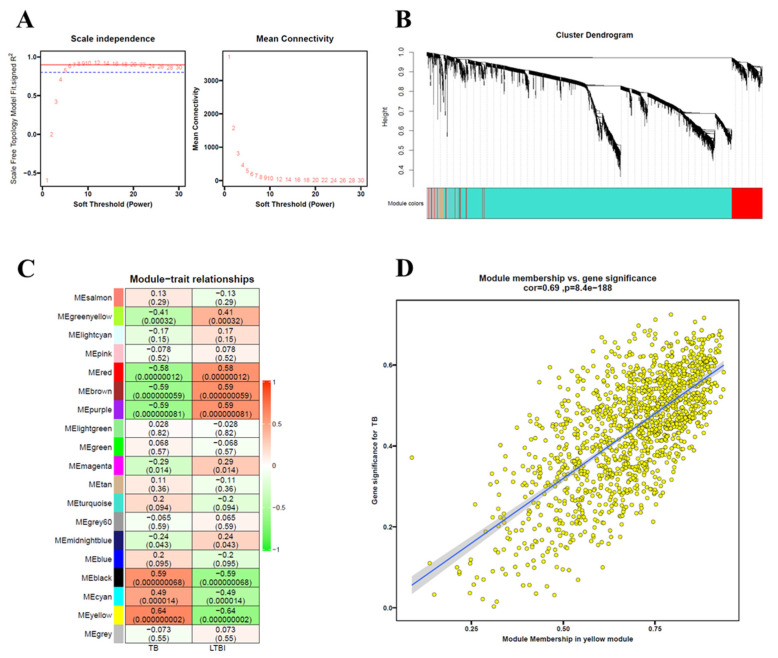
Identification of hub modules in GSE28623 dataset. (**A**) Scale-free topology model fit (R^2^ = 0.8) and mean connectivity against soft threshold power values (power, β = 7), assessing network independence and module stability. (**B**) The cluster dendrogram, illustrating hierarchical gene clustering with distinct module colors representing functional groups. The colored row below the dendrogram indicates modules as determined by the module cuttree height of 0.3. (**C**) Heatmap showing module–trait relationships, with colored bars on the left indicating different modules. Rows represent Pearson correlation coefficient and *p*-values between gene modules and TB/LTBI conditions. (**D**) Scatterplots of gene significance for TB (y-axis) vs. module membership (*x*-axis) of the yellow module, with a correlation of 0.69 and *p*-value of 8.4 × 10^−188^, suggesting strong association with disease traits.

WGCNA was performed using the GSE28623 dataset to identify key modules related to TB. A power of β = 7 was chosen as the soft thresholding value to construct a scale-free topology model fit (Figure 7A). After constructing a similar module cluster using a cuttree height of 0.3, a total of 19 modules were identified (Figure 7B). Among these modules, the yellow module exhibited the highest correlation with TB (Figure 7C). The correlation of the yellow module with gene significance for TB was 0.69 (*p*-value = 8.4 × 10^−188^) (Figure 7D).

### 3.8. Identification of FR-DEGs in the Yellow Module

**Figure 8:**
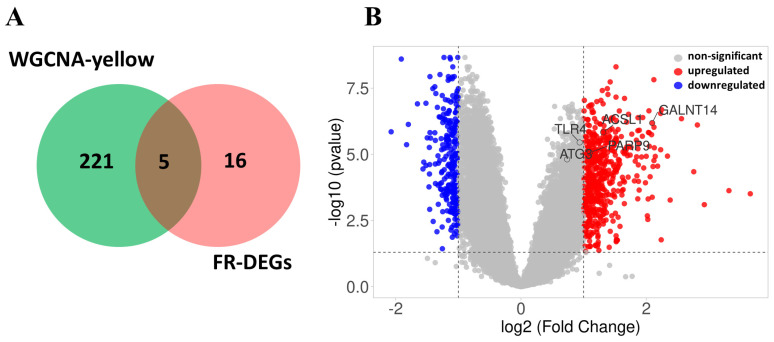
Selection of hub genes and volcano plot: (**A**) Venn diagram showing the overlap between WGCNA-yellow module genes (n = 226) and 21 FR-DEGs, resulting in the identification of five hub genes. (**B**) The volcano plot displays differential gene expression, highlighting significantly upregulated genes in red, downregulated genes in blue, and non-significant genes in gray, highlighting 5 hub genes.

The genes within the yellow module (n = 226) intersected with 21 FR-DEGs, resulting in the identification of five overlapping hub genes, *ACSL1*, *TLR4*, *PARP9*. *ATG3*, and *GALNT14*, which were found to be associated with TB (Figure 8A). However, *GALNT14* was removed from further validation analysis as it was not identified in the first three validation datasets. The volcano plot displays all of the deregulated genes, with the five hub genes highlighted (Figure 8B).

### 3.9. Correlation Between Hub Gene Expression and Immune Cell Infiltration Levels of GSE28623

**Figure 9:**
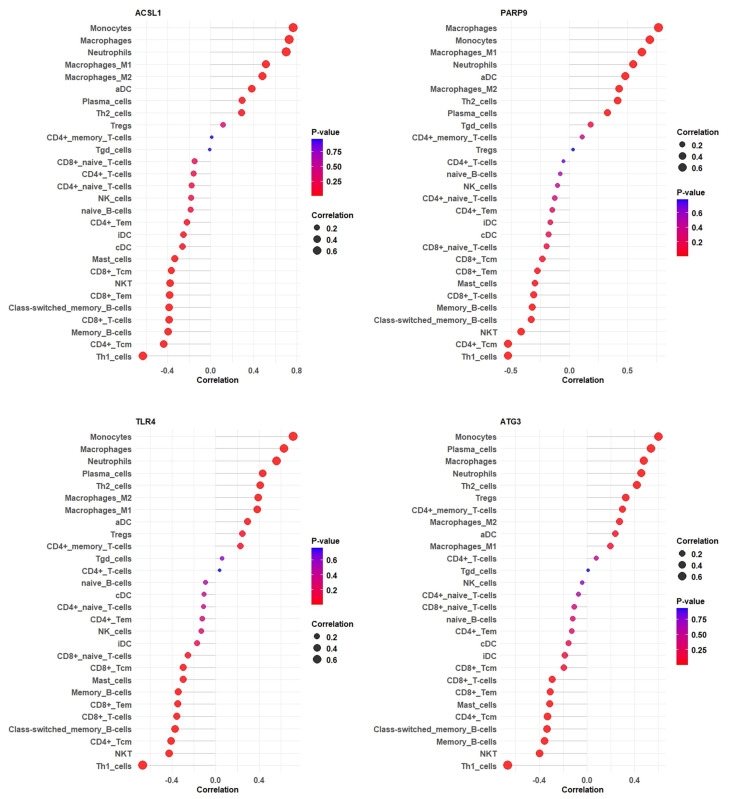
The lollipop plots illustrate the correlation of immune cell types with *ACSL1*, *PARP9*, *TLR4*, and *ATG3* expression, showing variations in correlation strength and statistical significance, which may provide insights into TB-related immune responses.

This study investigated the correlation between hub gene expression and immune cell infiltration levels, revealing significant associations. Specifically, the research examined the relationships between *ACSL1*, *PARP9*, *TLR4*, and *ATG3* gene expression and various immune cell types. The results indicated a negative correlation between *ACSL1* expression and Th1 cells, CD4+ T-cells, memory B-cells, CD8+ T-cells, class-switched memory B-cells, CD8+ Tem, NK T-cells, and CD8+ Tcm, while showing a positive correlation with monocytes, macrophages, neutrophils, and macrophages M1 and M2. Similarly, *PARP9* expression was negatively correlated with Th1 cells, CD4+ Tcm, NK T-cells, class-switched memory B-cells, memory B-cells, CD8+ T-cells, mast cells, CD8+ Tem, and CD8+ Tcm and positively correlated with macrophages, monocytes, macrophage M1, neutrophils, aDC, macrophage M2, and Th2 cells. *TLR4* expression was negatively correlated with Th1 cells, NK T-cells, CD4+ Tcm, class-switched memory B-cells, CD8+ T-cells, CD8+ Tem, memory B-cells, mast cells, CD8+ Tcm, and CD8+ naïve T-cells but positively correlated with monocytes, macrophages, neutrophils, plasma cells, Th2 cells, and macrophages M1 and M2. Finally, *ATG3* expression was negatively correlated with Th1 cells, NK T-cells, memory B-cells, class-switched memory B-cells, CD4+ Tcm, mast cells, CD8+ Tem, and CD8+ T-cells and positively correlated with monocytes, plasma cells, macrophages, neutrophils, Th2 cells, Tregs, CD4+ memory T-cells, macrophage M2, and aDC (Figure 9).

### 3.10. Validation and Diagnostic Value of Hub Genes in TB and LTBI

**Figure 10:**
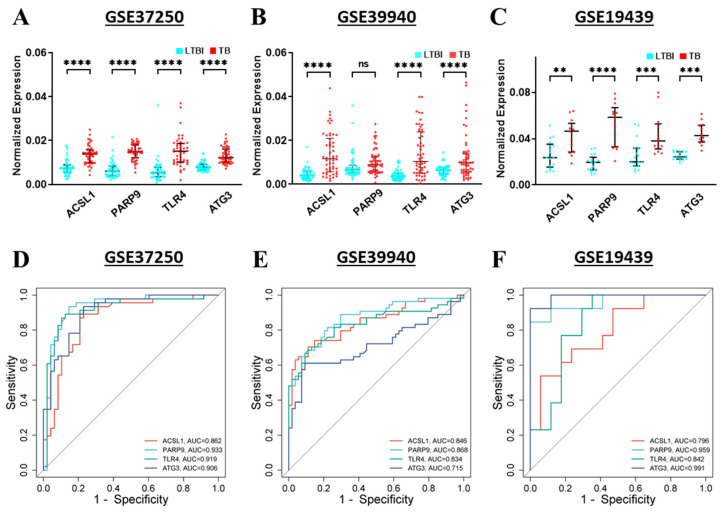
Validation of 4 hub genes in GSE37250, GSE39940, and GSE19439 datasets. (**A**–**C**) illustrate the expression levels of *ACSL1*, *PARP9*, *TLR4*, and *ATG3* in LTBI and TB samples, highlighting significant differences in gene regulation. (**D**–**F**) present the ROC analysis for *ACSL1*, *PARP9*, *TLR4*, and *ATG3* from the same datasets, demonstrating their diagnostic potential for TB based on high AUC values. ** *p* < 0.01, *** *p* < 0.001, **** *p* < 0.0001, ns—non-significant.

To validate gene expression, microarray datasets GSE37250, GSE39940, and GSE19439 were utilized. The TB group exhibited significantly higher expression levels of *ACSL1*, *PARP9*, *TLR4*, and *ATG3* genes compared to the LTBI group in the GSE37250 dataset (*p* < 0.0001) (Figure 10A). In the GSE39940 dataset, *ACSL1*, *TLR4*, and *ATG3* genes were significantly upregulated (*p* < 0.0001), while *PARP9* showed no significant difference in gene expression between TB and LTBI groups (Figure 10B). For the GSE19439 dataset, all four hub genes showed significant upregulation: *ACSL1* (*p* < 0.01), *PARP9* (*p* < 0.0001), *TLR4* (*p* < 0.001), and *ATG3* (*p* < 0.001) (Figure 10C).

ROC plot analysis was employed to confirm the diagnostic value of hub genes, specifically *ACSL1*, *PARP9*, *TLR4*, and *ATG3*, in distinguishing TB from LTBI. In the GSE37250 cohort, the AUC values were 0.862 for *ACSL1*, 0.933 for *PARP9*, 0.919 for *TLR4*, and 0.906 for *ATG3* (Figure 10D). For the GSE39940 cohort, the AUC values were 0.846 for *ACSL1*, 0.868 for *PARP9*, 0.834 for *TLR4*, and 0.715 for *ATG3* (Figure 10E). Similarly, in the GSE19439 cohort, the AUC values were 0.796 for *ACSL1*, 0.959 for *PARP9*, 0.842 for *TLR4*, and 0.991 for *ATG3* (Figure 10F). The results indicate that the four hub gene signatures have potential as promising biomarkers to differentiate TB from LTBI. The analysis demonstrated high sensitivity and specificity, 89.1% and 94.1% for *ACSL1*, 93.5% and 100% for *PARP9*, 100% and 87.5% for *TLR4*, and 93.5% and 100% for *ATG3*, respectively, across the three datasets.

The PCA plots of all three datasets illustrate the distribution of LTBI and TB groups based on principal components. In the GSE37250 dataset, PC1 explains 58.8% of the variance, while PC2 accounts for 25.8%, providing a combined variance of 84.6%. In the GSE39940 dataset, PC1 explains 57.4% of the variance, while PC2 accounts for 29.3%, resulting in a combined variance of 86.7%. In the GSE19439 dataset, PC1 explains 54.2% of the variance, while PC2 accounts for 29.4%, giving a combined variance of 83.6%. Despite the high explained variance, the overlap between the LTBI and TB groups suggests shared molecular traits or insufficient discriminatory features in the principal components (Figure S1).

Similarly, the heatmaps of all three datasets also showed gene expression patterns in LTBI and TB samples, with blue indicating lower and red higher expression, while hierarchical clustering highlights group similarities. The mixed clustering suggests overlapping molecular traits (Figure S2).

**Figure 11:**
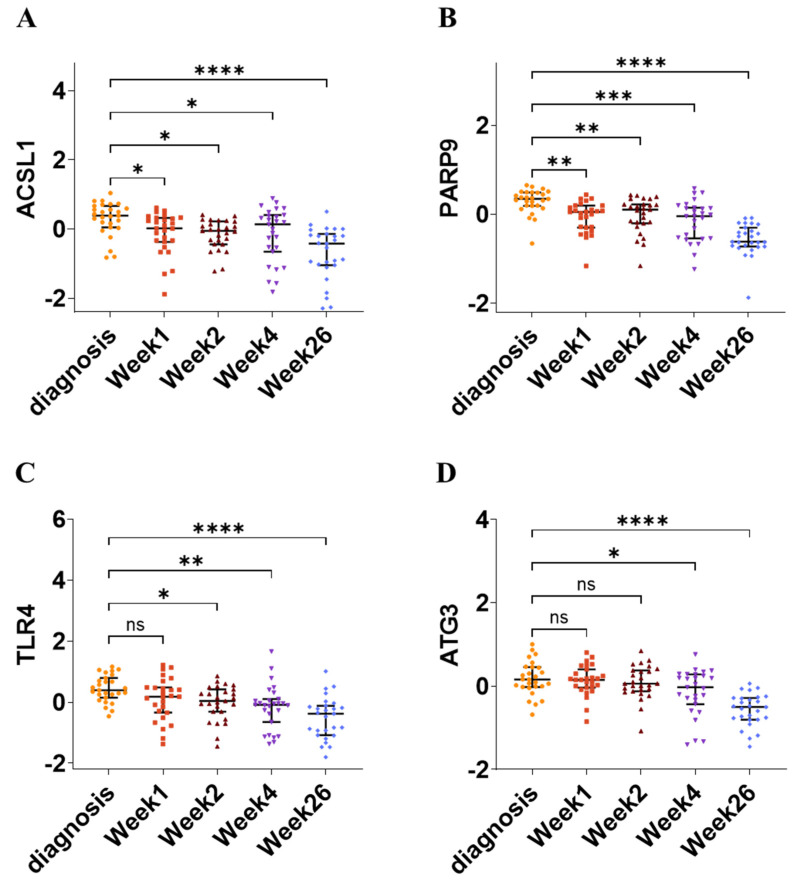
Association of hub gene signatures with anti-TB treatment efficacy at 5 distinct time points in GSE31348 dataset. (**A**–**D**) The expression levels of *ACSL1*, *PARP9*, *TLR4*, and *ATG3* change significantly across different time points during TB treatment (diagnosis, week 1, week 2, week 4, and week 26), highlighting their dynamic regulation in disease progression. In each scatter plot, the central horizontal line represents the median, while the endpoints indicate the first quartile (Q1) and third quartile (Q3), defining the interquartile range (IQR). The endpoints of the central vertical line mark the minimum and maximum values. * *p* < 0.05, ** *p* < 0.01, *** *p* < 0.001, **** *p* < 0.0001, ns—non-significant.

To evaluate the prognostic value of the four hub genes (*ACSL1*, *PARP9*, *TLR4*, and *ATG3*), the GSE31348 gene expression dataset, containing 135 blood samples from 27 pulmonary TB patients assayed at diagnosis and after 1, 2, 4, and 26 weeks of TB treatment, was utilized. The hub gene signature’s expression was highest at diagnosis and significantly decreased during anti-TB treatment (Figure 11). The PCA plot illustrates the distribution of data points across PC1 (43.5% variance) and PC2 (18.5% variance), with colored ellipses representing different time points, showing clustering patterns and sequential alterations. The heatmap displays gene expression levels for *ACSL1*, *PARP9*, *TLR4*, and *ATG3*, revealing dynamic changes during anti-TB treatment, as indicated by changing color gradients across different time points (Figure S3). This indicates that the four hub gene panel is associated with anti-TB treatment effectiveness and could serve as valuable drug targets for TB diagnosis.

## 4. Discussion

Tuberculosis continues to pose a significant global health challenge, primarily affecting the lungs but capable of spreading to other organs [33]. Traditional TB diagnosis relies heavily on acid-fast bacillus (AFB) sputum smear microscopy, which has limitations due to its low sensitivity and can result in undiagnosed cases and treatment delays [34]. The immune response, particularly T-cell-mediated adaptive immunity, plays a vital role in combating *Mtb* infection [35,36]. Recent studies have highlighted the connection between imbalances in ferroptotic cell death and impaired immune responses in TB pathology [37]. Ferroptosis, a form of regulated cell death, is being explored as a potential therapeutic target, with the identification of ferroptosis-related markers considered crucial for improving TB diagnosis, treatment, and prognosis [38].

Necrotic cell death during TB infection is detrimental to the host, facilitating the spread of *Mtb*. Ferroptosis plays a significant role in this process, characterized by iron accumulation and lipid peroxidation [8,39]. GPX4 has been identified as crucial in preventing ferroptosis triggered by *Mtb* infection [40]; however, the molecular mechanism underlying ferroptosis in TB remains not fully understood. High-throughput technologies, such as microarrays, have become essential tools for studying gene expression changes in TB and other diseases.

This study aimed to identify key deregulated genes in TB infection through the analysis of the GSE28623 dataset from GEO using a bioinformatics approach. sPLS-DA demonstrated an overlap between TB and LTBI groups (Figure 2). Immune cell infiltration analysis revealed significantly higher expression and composition of various immune cells in the TB group, including plasma cells, regulatory T-cells, monocytes, macrophages, macrophages M1 and M2, activated DC, Th2 cells, and neutrophils, which is consistent with earlier findings [41,42,43,44,45,46,47] (Figure 3).

GSEA revealed that numerous genes associated with ferroptosis are enriched in TB, suggesting their crucial role in regulating the immune response in TB (Figure 4), corroborating the findings of Zhang et al. [14]. GEO2R analysis identified 599 DEGs between TB and LTBI groups, with 371 upregulated and 228 downregulated. To find ferroptosis-related DEGs (FR-DEGs), we intersected 502 FRGs with the 599 DEGs, resulting in 21 FR-DEGs (Figure 5A and Table 1). Using the STRING database, a predicted protein–protein interaction network was constructed for these genes and found good interaction among them (Figure 5B). Furthermore, correlation analysis revealed a synergistic relationship among *ACSL1*, *PARP9*, *TLR4*, and *ATG3*, which were significantly upregulated in TB patients and strongly correlated with each other (Figure 5C).

The integration of GO, KEGG, and WikiPathways enrichment analyses provides a robust framework for elucidating the biological significance of the identified FR-DEGs. GO and KEGG pathway enrichment analyses revealed that the 21 FR-DEGs were significantly enriched in ferroptosis, autophagy, and fatty acid biosynthesis (Figure 6A). Additionally, WikiPathways analysis confirmed a strong association between ferroptosis and TB infection, highlighting its crucial role in the onset, progression, and regulation of TB (Figure 6B).

Weighted Gene Co-Expression Network Analysis (WGCNA) identified a highly significant yellow module containing key genes dysregulated in TB (Figure 7). By intersecting the yellow module genes with the 21 ferroptosis-related differentially expressed genes (FR-DEGs), we identified five hub genes, all of which were upregulated in the TB group (Figure 8). Correlation analysis of four hub genes with immune cell infiltration revealed that most immune cells, including macrophages, monocytes, and neutrophils, were significantly correlated with these genes (Figure 9).

Validation of the four hub genes in independent datasets (GSE37250, GSE39940, GSE19439, and GSE31348) further confirms the reliability of the results. All four genes exhibit significant upregulation in the TB group as compared to the LTBI group across the GSE37250, GSE39940, and GSE19439 datasets (Figure 10A–C). Receiver Operating Characteristic (ROC) curve analyses in these three datasets demonstrated strong diagnostic performance, with area under the curve (AUC) values supporting the hub genes’ relevance as TB diagnostic biomarkers, achieving peak sensitivity and specificity of 89.1% and 94.1% for *ACSL1*, 93.5% and 100% for *PARP9*, 100% and 87.5% for *TLR4*, and 93.5% and 100% for *ATG3* across the datasets (Figure 10D–F). Additional validation in the GSE31348 dataset revealed the downregulation of all four hub genes with anti-TB treatment (Figure 11). The PCA plot reveals sequential shifts in clustering patterns, with high combined variance reflecting progressive changes over time. Additionally, the heatmap shows a decrease in the expression of *ACSL1*, *PARP9*, *TLR4*, and *ATG3* during anti-TB treatment, suggesting these genes as potential biomarkers for disease monitoring and therapeutic interventions.

Several studies have demonstrated a strong relationship between ferroptosis and tuberculosis, identifying key biomarkers that play a crucial role in disease progression and immune regulation. A bioinformatics study utilizing blood microarray transcriptional datasets identified three ferroptosis-related genes-*CHMP5*, *SAT1*, and *ZFP36*-as potential diagnostic biomarkers for tuberculosis (TB) [13]. Another study by Liang et al. found significant upregulation of SOCS1 in TB patients which decreases after anti-TB treatment [48]. A recent study identified nine ferroptosis-related genes, *MAPK14*, *EGLN2*, *IDO1*, *USP11*, *SCD*, *CBS*, *PARP8*, *PARP16*, and *CDC25A*, associated with TB, which are considered probable biomarkers for distinguishing latent TB infection from active TB in children [12]. A recent study by Zhang et al. (2025) demonstrated that ferroptosis plays a critical role in TB pathogenesis and identified *IL1B*, *PTGS2*, *TNFAIP3*, *HMOX1*, *SOCS1*, *CD82*, and *NUPR1* as key genes linked to *Mtb*-induced ferroptosis [14]. Another recent study suggests that monocyte differentiation trajectories, transcription factor dysregulation, and impaired ferroptosis play a critical role in TB progression, with CEBPB, CORO1A, IRF9, MEF2C, MICU1, PRR5, MIF, and LGALS9 identified as key molecular regulators [49].

This study identified four ferroptosis-related hub gene signatures: *ACSL1*, *PARP9*, *TLR4*, and *ATG3*. *ACSL1* (acyl-CoA synthetase long-chain family member 1), an isoenzyme of long-chain acyl-CoA synthetase (ACSL), catalyzes the conversion of long-chain fatty acids into their active acyl-CoA forms, which are used for cellular lipid synthesis and degradation via β-oxidation [50]. *ACSL1* is important for α-eleostearic acid (αESA)-induced ferroptosis, as it helps in the production of neutral lipids, such as diacylglycerols (DAGs) and triacylglycerols (TAGs) [51], and sensitizes cells to ferroptosis by accumulating αESA in TAGs. Recent studies have confirmed *ACSL1* as a promoter of ferroptosis [52]. In human peripheral blood mononuclear cell (PBMC)-derived macrophages infected with *Mtb*, *ACSL1* expression was elevated, accompanied by increased lipid droplet formation [53]. In this study, *ACSL1* expression levels were consistent with findings from previous research.

PARPs are a family of proteins involved in various pathologies, including metabolic disorders, stress responses, cancers, inflammatory responses, and viral and bacterial infections [54]. *PARP9* [poly (ADP-ribose) polymerase 9], a member of the PARP family, enhances proinflammatory cytokine production in response to IFN-γ stimulation by promoting STAT1 phosphorylation [55]. In TB, *PARP9* is significantly upregulated and associated with an increased risk of infection [56]. Although the *PARP9* gene is hypomethylated in TB patients [57], it was identified as part of a three-gene signature predicting progression to active TB in primates [58]. These finding collectively suggest that *PARP9* is a key regulator of TB, consistent with our results.

Toll-like receptors (TLRs) are pattern recognition receptors (PRRs) which function in innate immunity. *TLR4* (Toll-like receptor 4), primarily expressed on the surface of immune cells such as macrophages, dendritic cells, and monocytes, signals through both MyD88-dependent and MyD88-independent pathways [59]. *TLR4* plays a critical role in the anti-TB immune response by recognizing *Mtb* and its components, thereby triggering innate immune responses [60]. During ferroptosis, HMGB1 released by M2 macrophages interacts with *TLR4* on M1 macrophages, activating STAT3 signaling in M1 macrophages and contributing to the inflammatory response [61]. Recent studies indicate that activation of inflammatory pathways, including multiple inflammation-related signaling cascades, can induce ferroptosis. [62]. Our study further supports the upregulation and potential involvement of *TLR4* in ferroptosis during TB infection.

Autophagy-related protein 3 (*ATG3*) plays a critical role in regulating autophagy during cell death [63]. By controlling autophagy, *ATG3* facilitates the clearance of infections. *Mtb*, in both live and virulent forms, significantly increases miR-155 expression in dendritic cells. By binding to the 3′-UTR of *ATG3*, MiR-155 inhibits *ATG3* translation, thereby suppressing autophagy and promoting *Mtb* survival [64]. In our study, *ATG3* was upregulated, consistent with previous research suggesting that *ATG3* and autophagy are essential for ferroptosis.

This study has several limitations. First, we relied on publicly available datasets for bioinformatics analysis, which may limit the scope of the findings. Second, the results were not validated through animal experiments or clinical studies, so they may not fully capture the complex biological processes in living organisms. Third, we analyzed only bulk RNA sequencing data for gene expression, without assessing corresponding protein expression levels. Finally, the analysis included data from tuberculosis (TB) patients and individuals with latent TB infection (LTBI) but did not incorporate data from healthy controls.

## 5. Conclusions

Our study highlights the significant role of ferroptosis in the pathogenesis of tuberculosis. Through comprehensive bioinformatics analyses, we identified and validated four hub genes-*ACSL1*, *PARP9*, *TLR4*, and *ATG3*-as key biomarkers associated with TB infection, with potential as diagnostic and therapeutic targets. These findings provide a foundation for future research aimed at developing targeted therapies for TB by modulating ferroptosis-related pathways. Ultimately, this work has the potential to enhance diagnostic accuracy and improve treatment outcomes for patients with TB.

## Figures and Tables

**Table 1:** List of 21 FR-DEGs.

Gene Symbol	Gene Title	−log10 (*p*-Value)	log_2_ (Fold Change)	UP/DOWN
*GALNT14*	Polypeptide N-acetylgalactosaminyltransferase 14	6.18	2.09	UP
*ACSL1*	acyl-CoA synthetase long-chain family member 1	5.83	1.31	UP
*CISD2*	CDGSH iron sulfur domain 2	4.96	1.31	UP
*LCN2*	lipocalin 2	3.44	1.31	UP
*PROK2*	prokineticin 2	3.87	1.21	UP
*BACH1*	BTB domain and CNC homolog 1	4.55	1.18	UP
*ACSL4*	acyl-CoA synthetase long-chain family member 4	4.13	1.16	UP
*CREB5*	cAMP responsive element binding protein 5	5.37	1.15	UP
*SLC40A1*	solute carrier family 40 member 1	5.74	1.15	UP
*ATG3*	autophagy related 3	4.20	1.11	UP
*PARP9*	Poly (ADP-ribose) polymerase family member 9	5.06	1.10	UP
*MAPK14*	mitogen-activated protein kinase 14	5.18	1.10	UP
*TLR4*	toll-like receptor 4	4.92	1.10	UP
*PTGS2*	prostaglandin-endoperoxide synthase 2	2.46	1.09	UP
*ATF3*	activating transcription factor 3	3.31	1.09	UP
*GCLC*	glutamate-cysteine ligase catalytic subunit	3.84	1.08	UP
*GLRX5*	glutaredoxin 5	4.38	1.07	UP
*CHMP5*	charged multivesicular body protein 5	4.21	1.05	UP
*GABARAPL2*	GABA type A receptor associated protein like 2	5.27	1.05	UP
*TP53*	tumor protein p53	3.29	−1.00	DOWN
*ALOX15*	arachidonate 15-lipoxygenase	2.25	−1.26	DOWN

## Data Availability

The data presented in this study using the GSE28623, GSE37250, GSE39940, GSE19439, and GSE31348 datasets are publicly available in the NCBI Gene Expression Omnibus database (GEO, http://www.ncbi.nlm.nih.gov/geo, accessed on 18 April 2024).

## Supplementary Materials

The following supporting information can be downloaded at: https://www.mdpi.com/article/10.3390/genes16060716/s1, Figure S1: PCA plots of three validation sets showing the distribution of patients using 4 hub gene signatures. Figure S2: Heatmaps of three validation sets showing the gene expression profiles of 4 hub gene signatures. Figure S3: PCA plot and heatmap of GSE31348 validation set showing the distribution of patients and the expression profiles of 4 hub gene signatures.
