# Supplementary material for: Bioinformatics-Driven Identification of Ferroptosis-Related Gene Signatures Distinguishing Active and Latent Tuberculosis
Source: Genes (Basel). 2025 Jun 18;16(6):716. doi: 10.3390/genes16060716 (PMC12192361; doi:10.3390/genes16060716)
Supplement: Supplementary file 1 [file genes-16-00716-s001.zip › genes-3666033-supplementary.pptx]

## Slide 1
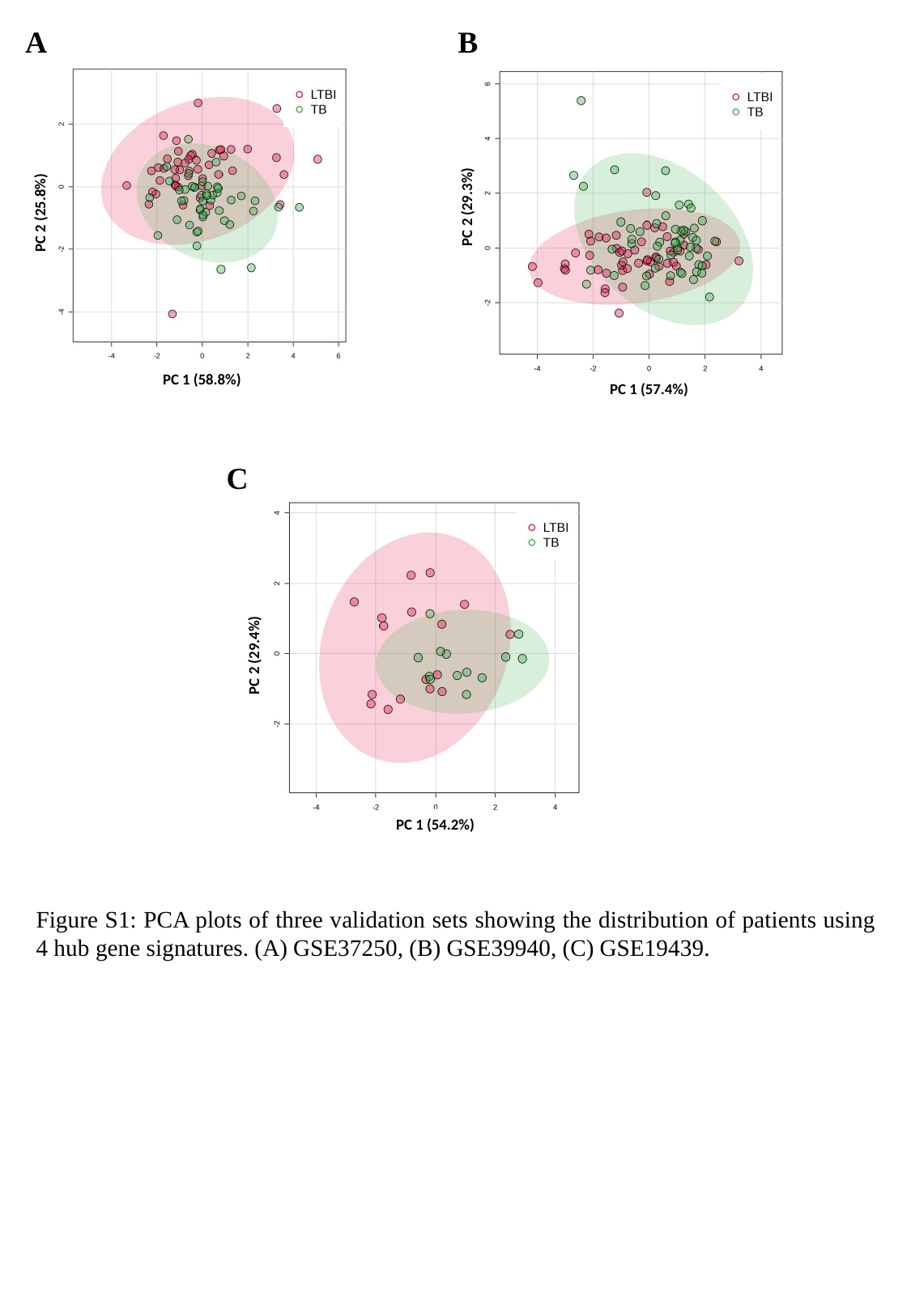

A
B
PC 2 (29.3%)
PC 1 (57.4%)
PC 2 (25.8%)
PC 1 (58.8%)
C
PC 2 (29.4%)
PC 1 (54.2%)
Figure S1: PCA plots of three validation sets showing the distribution of patients using 4 hub gene signatures. (A) GSE37250, (B) GSE39940, (C) GSE19439.

## Slide 2
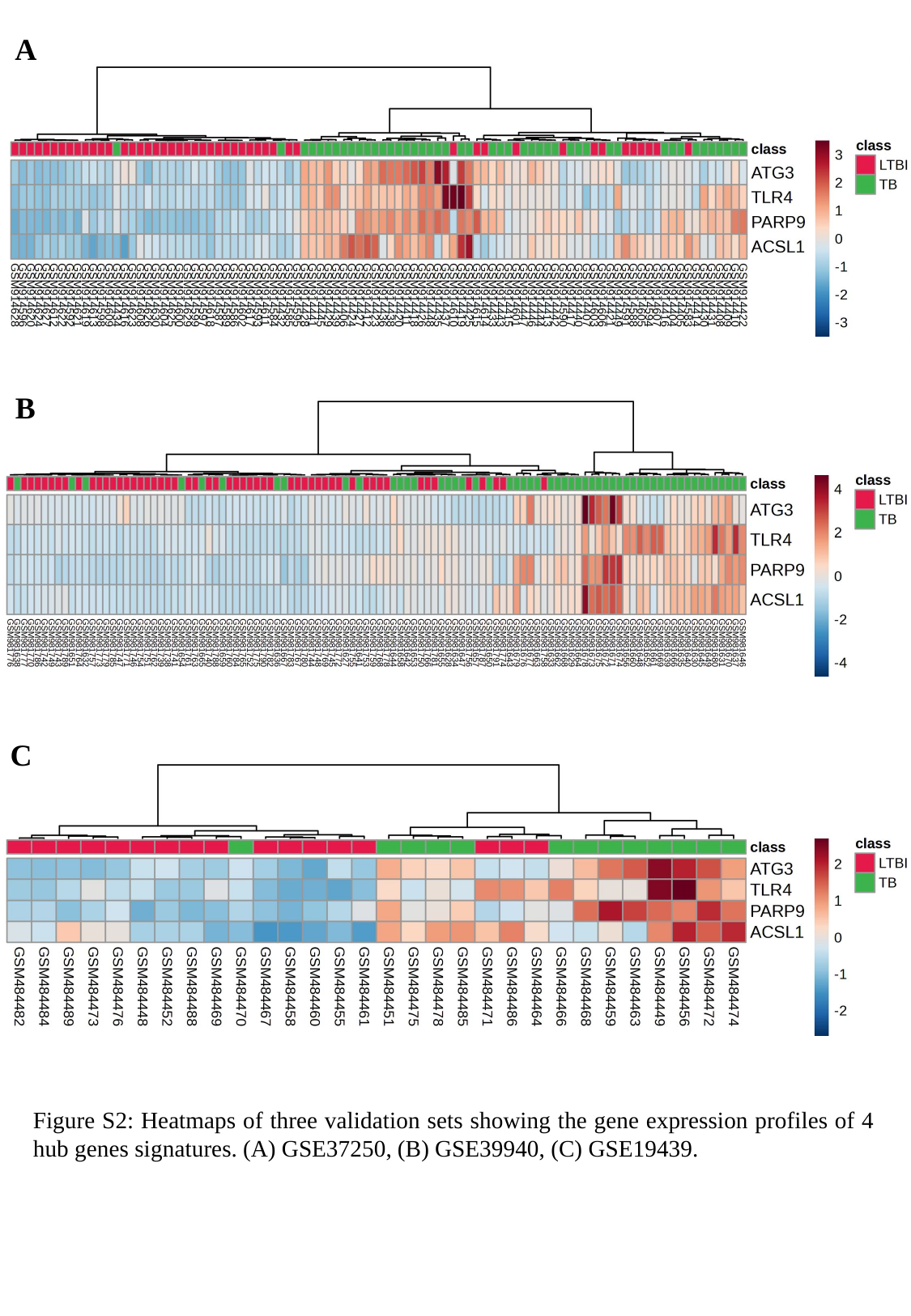

A
B
C
Figure S2: Heatmaps of three validation sets showing the gene expression profiles of 4 hub genes signatures. (A) GSE37250, (B) GSE39940, (C) GSE19439.

## Slide 3
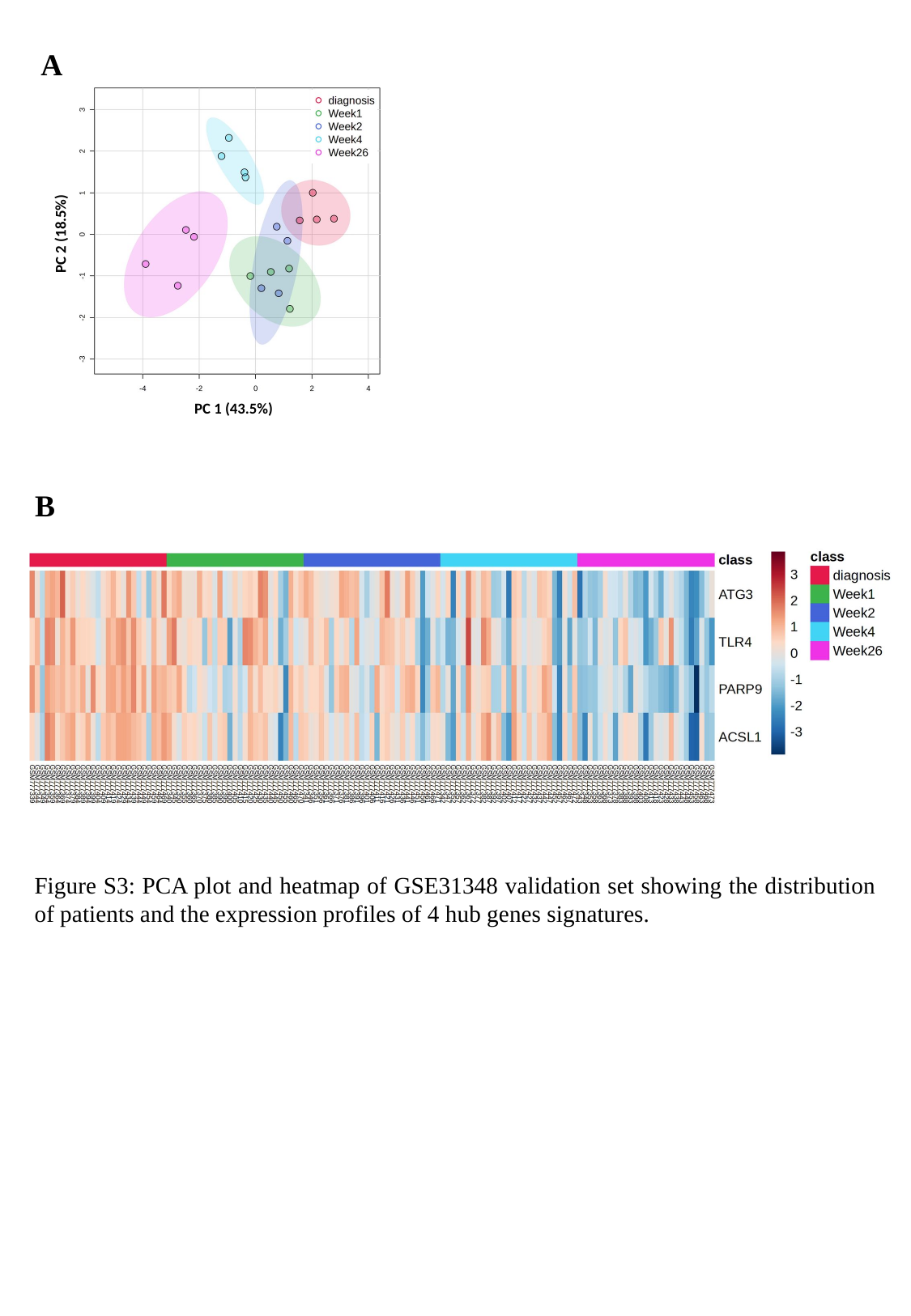

A
PC 2 (18.5%)
PC 1 (43.5%)
B
Figure S3: PCA plot and heatmap of GSE31348 validation set showing the distribution of patients and the expression profiles of 4 hub genes signatures.
